# Genetic Mapping of QTLs Controlling Fatty Acids Provided Insights into the Genetic Control of Fatty Acid Synthesis Pathway in Peanut (*Arachis hypogaea* L.)

**DOI:** 10.1371/journal.pone.0119454

**Published:** 2015-04-07

**Authors:** Ming Li Wang, Pawan Khera, Manish K. Pandey, Hui Wang, Lixian Qiao, Suping Feng, Brandon Tonnis, Noelle A. Barkley, David Pinnow, Corley C. Holbrook, Albert K. Culbreath, Rajeev K. Varshney, Baozhu Guo

**Affiliations:** 1 Plant Genetics Resources Conservation Unit, US Department of Agriculture-Agricultural Research Service, Griffin, Georgia, United States of America; 2 Crop Protection and Management Research Unit, US Department of Agriculture-Agricultural Research Service, Tifton, Georgia, United States of America; 3 International Crops Research Institute for the Semi-Arid Tropics (ICRISAT), Hyderabad, Telangana, India; 4 Department of Plant Pathology, University of Georgia, Tifton, Georgia, United States of America; 5 Fujian Agriculture and Forestry University, Fuzhou, Fujian, China; 6 College of Life Science, Qingdao Agricultural University, Qingdao, Shandong, China; 7 College of Bioscience and Biotechnology, Qiongzhou University, Sanya, Hainan, China; 8 Crop Genetics and Breeding Research Unit, US Department of Agriculture-Agricultural Research Service, Tifton, Georgia, United States of America; National Institute of Plant Genome Research, INDIA

## Abstract

Peanut, a high-oil crop with about 50% oil content, is either crushed for oil or used as edible products. Fatty acid composition determines the oil quality which has high relevance to consumer health, flavor, and shelf life of commercial products. In addition to the major fatty acids, oleic acid (C18:1) and linoleic acid (C18:2) accounting for about 80% of peanut oil, the six other fatty acids namely palmitic acid (C16:0), stearic acid (C18:0), arachidic acid (C20:0), gadoleic acid (C20:1), behenic acid (C22:0), and lignoceric acid (C24:0) are accounted for the rest 20%. To determine the genetic basis and to improve further understanding on effect of FAD2 genes on these fatty acids, two recombinant inbred line (RIL) populations namely S-population (high oleic line ‘SunOleic 97R’ × low oleic line ‘NC94022’) and T-population (normal oleic line ‘Tifrunner’ × low oleic line ‘GT-C20’) were developed. Genetic maps with 206 and 378 marker loci for the S- and the T-population, respectively were used for quantitative trait locus (QTL) analysis. As a result, a total of 164 main-effect (M-QTLs) and 27 epistatic (E-QTLs) QTLs associated with the minor fatty acids were identified with 0.16% to 40.56% phenotypic variation explained (PVE). Thirty four major QTLs (>10% of PVE) mapped on five linkage groups and 28 clusters containing more than three QTLs were also identified. These results suggest that the major QTLs with large additive effects would play an important role in controlling composition of these minor fatty acids in addition to the oleic and linoleic acids in peanut oil. The interrelationship among these fatty acids should be considered while breeding for improved peanut genotypes with good oil quality and desired fatty acid composition.

## Introduction

Peanut or groundnut (*Arachis hypogaea* L., 2*n* = 4*x* = 40) is a major oil crop along with soybean and cotton. China, India, and the United States are the leading producers contributing about 70% of the world peanut crop. Peanut seeds are used for extracting cooking oil by crushing, consumed as fresh/boiled/roasted seeds, confectionary preparations, flour and butter. Peanut seeds contain around 25% protein, 50% oil and many useful secondary plant metabolites such as flavonoid, folic acid, tocopherols and resveratrol [1, 2]. Market preference exists for peanuts with both low as well as high oil content. Genotypes with high oil content and high oleate traits are preferred for oil crushing industry while genotypes with low oil content and high oleate traits are preferred by consumers due to health advantage and longer shelf life of peanut products. Altering the composition of fatty acids in the seed oil is an important breeding objective for peanut cultivar development.

A monounsaturated fatty acid (MUFA), such as oleic acid (C18:1), is about 47% in normal and up to 80% in high oleic peanut lines and is associated with several human health benefits such as decreasing the risk of cardiovascular disease (CVD) by reducing the levels of serum low-density lipoproteins (LDL) cholesterol and maintaining the levels of high-density lipoproteins (HDL). The MUFA also helps in hampering the development of adrenoleukodystrophy (ALD) and reversing inhibitory effects of insulin production [3, 4, 5]. The next major fatty acid, a polyunsaturated fatty acid (PUFA) i.e., linoleic acid (C18:2), is known for its vulnerability to oxidative rancidity, making it undesirable for human intake as it becomes thermodynamically unstable when heated at high temperature [6]. This instability leads to formation of *trans* fatty acid which has detrimental effect on human health as it causes cardiovascular disease (CVD).

The oleic acid (C18:1) and linoleic acid (C18:2) accounts for about 80% of peanut oil while the minor saturated fatty acids (SFA) such as palmitic acid (C16:0), stearic acid (C18:0), arachidic acid (C20:0), gadoleic acid (C20:1), behenic acid (C22:0), and lignoceric acid (C24:0) account for the remaining 20% fatty acid [7]. Although gadoleic acid is present in trivial quantity, it is considered to be a healthy fatty acid found conspicuous in fish oils such as cod liver oil and in breast milk [8]. Saturated fatty acids (SFA) have been found to increase the blood LDL cholesterol level. The ratio of UFAs (oleic and linoleic acid) to SFAs (palmitic acid, stearic acid, arachidic acid, behenic acid, and lignoceric acid) in peanut oil is very high while the amount of SFAs in peanut oil is considerably lower than the butter, coconut oil and palm oil, thereby making peanut oil as one of the healthy alternative as a cooking oil [9]. However, not all SFAs are considered to be unhealthy, studies have shown that stearic acid (C18:0) had no effect on total cholesterol levels in contrast to palmitic acid (C16:0). Stearic acid (C18:0) has been a healthy substitute for *trans* fatty acid in food manufacturing [10]. Furthermore, a recent study revealed that the circulating high levels of fatty acids such as stearic (C18:0), arachidic (C20:0), behenic (C22:0) and lignoceric (C24:0) acid lowers the risk of atria fibrillation as compared to palmitic acid (C16:0) [11]. Interestingly, lignoceric acid (C24:0) in a minor quantity was found to be essential for development and maintenance of brain function [12].

Efforts for improving the peanut oil quality have yielded the identification of a high oleic acid peanut lines (F435-2-1 and F435-2-2) comparable to that in olive oil [13]. The high oleic acid content was obtained on the cost of lower linoleic acid. Genetics studies revealed that the high oleic acid is controlled by two homozygous recessive mutant genes *FAD2A* and *FAD2B* [14]. However, the effect of genetic regulation of *FAD2* genes over SFAs is not known. Nevertheless, three recent studies using germplasm collection as well as biparental populations indicated that there was a positive correlation between oleic acid (C18:1) with gadoleic acid (C20:1) and lignoceric acid (C24:0); and a negative correlation of oleic (C18:1) with stearic acid (C18:0), palmitic acid (C16:0), linoleic acid (C18:2), arachidic acid (C20:0) and behenic acid (C22:0) [2, 15, 16].

In recent years, although the understanding of the fatty acid biosynthetic pathway in crop plants has improved rapidly [17, 18], but the basic genetic control behind the regulation of fatty acid synthesis is still very limited [19]. Studies towards deciphering the molecular basis of *FAD2* genes revealed that it encodes microsomal oleoyl-PC desaturase or Δ^12^ fatty acid desaturase [20]. Further, the deployment of genomics tools such as linked markers promises to increase breeding efficiency of conventional breeding approaches leading to the rapid development of improved cultivars [21, 22, 23]. In this context, Pandey et al. [24] reported the relationship of *FAD2* genes with peanut oil quality and suggested that *FAD2B* contributed higher phenotypic variance for oleic (C18:1) and linoleic (C18:2) acids than the *FAD2A* alleles. However, so far no quantitative trait locus (QTL) study has been undertaken for the minor fatty acids except oleic (C18:1) and linoleic (C18:2) acids in peanuts.

In this study, two segregating populations with two years phenotypic data were used to identify QTLs controlling oil quality traits in peanut. The results obtained could improve our current understanding and provide evidences on the following aspects: (i) correlations among different saturated and unsaturated fatty acids, (ii) QTLs controlling these fatty acids and (iii) identification of consistent QTLs for these fatty acids, and (iv) effect of *FAD2A* and *FAD2B* mutant alleles on these fatty acids.

## Results

### Phenotypic variation and correlation between different fatty acids

**Fig 1:**
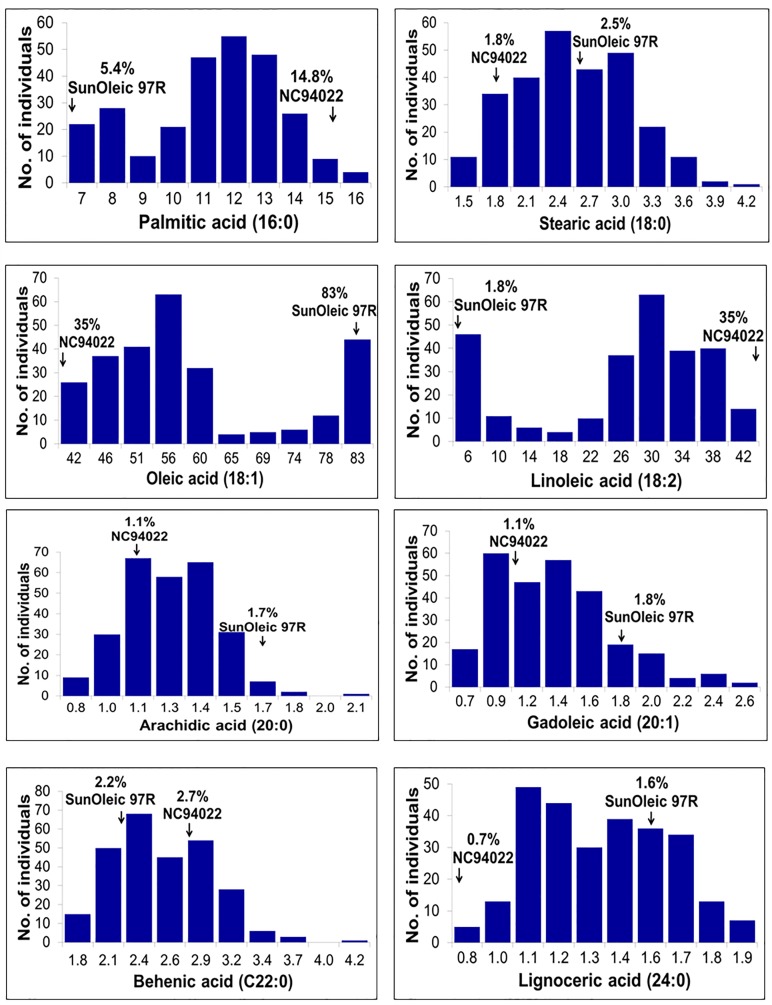
Phenotypic distribution of eight fatty acids in the S- population. The x-axis shows the range of percentage of average of two years (2010–2011) of fatty acids and the y-axis represents the number of individuals in the RIL population.

**Fig 2:**
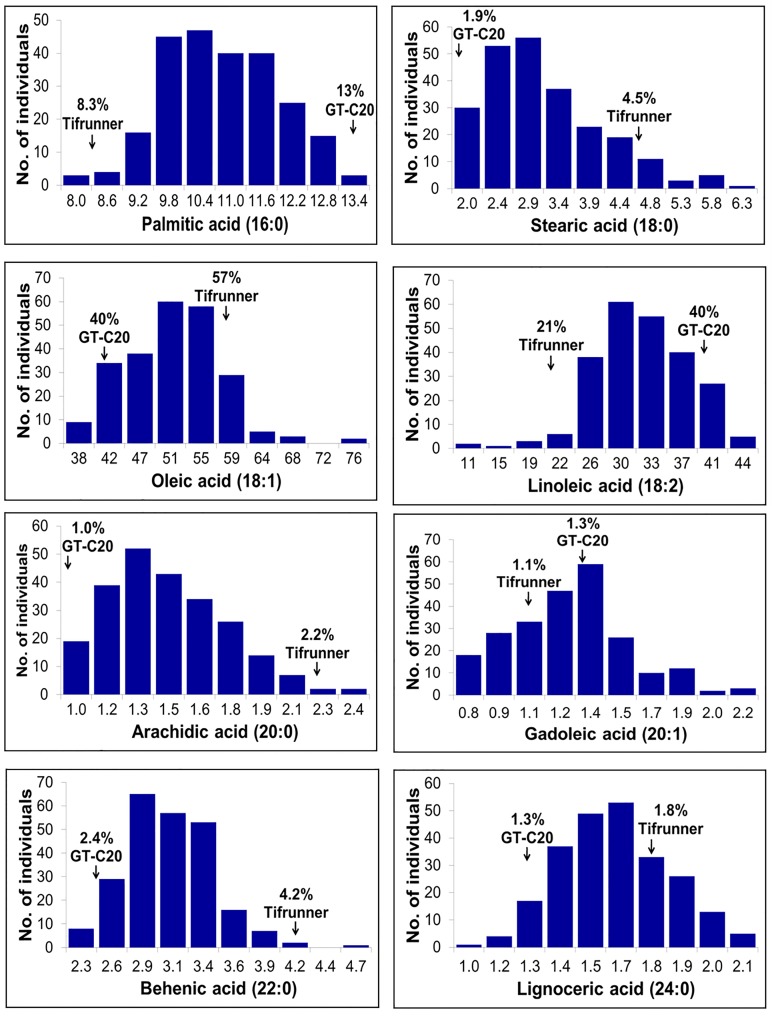
Phenotypic distribution of eight fatty acids in the T- population. The x-axis shows the range of percentage of average of two years (2010–2011) of fatty acids and the y-axis represents the number of individuals in the RIL population.

Phenotyping data for two seasons were generated for all the six SFAs (palmitic acid, stearic acid, arachidic acid, gadoleic acid, behenic acid, and lignoceric acid) along with UFAs (oleic and linoleic acid) on all four parents and the complete set of RILs of the S- (Fig 1) and T-population (Fig 2). Significant phenotypic variation was observed for different UFAs and SFAs in both the RIL populations (Figs 1 and 2). Phenotypic analysis revealed that the fitted curves were found to be either normally distributed (stearic acid, lignoceric acid), skewed (gadoleic acid, behenic acid), or multimodal (palmitic acid, oleic acid, linoleic acid). Pairwise correlation analysis on these fatty acids in both the RIL populations showed similar trend in trait association (Table 1). Significant negative correlation was observed between oleic acid and palmitic acid (r = -0.922 in the S- and -0.838 in the T-population) as well as between oleic acid and linoleic acid (r = -0.998 in the S- and -0.988 in the T-population). A positive correlation was found between oleic acid and gadoleic acid (r = 0.677 in the S- and 0.433 in the T-population). Stearic acid was positively and negatively correlated, respectively, with arachidic acid (r = 0.883 in the S- and 0.969 in the T-population) and gadoleic acid (r = -0.705 in the S- and -0.828 in the T-population). Lignoceric acid was positively correlated with gadoleic acid (r = 0.705 in the S- and 0.790 in the T-population) and behenic acid (r = 0.727 in the S- and 0.251 in the T-population).

**Table 1:** Phenotypic pairwise correlation among eight fatty acid traits in the S- and T-population.

Different fatty acids	Oleic acid (C18:1)	Linoleic acid (C18:2)	Palmitic acid (C16:0)	Stearic acid (C18:0)	Arachidic acid (C20:0)	Gadoleic acid (C20:1)	Behenic acid (C22:0)
**S- population (SunOleic 97R × NC94022)**							
Linoleic acid (C18:2)	-0.998***						
Palmitic acid (C16:0)	-0.922***	0.917***					
Stearic acid (C18:0)	-0.258**	0.231**	0.182*				
Arachidic acid (C20:0)	-0.31**	0.28**	0.105	0.883***			
Gadoleic acid (C20:1)	0.677***	-0.669***	-0.725***	-0.705***	-0.511***		
Behenic acid (C22:0)	-0.217*	0.196*	-0.09	0.158	0.576***	0.166	
Lignoceric acid (C24:0)	0.158	-0.161	-0.402**	-0.406**	-0.014	0.705***	0.727***
**T- population (Tifrunner × GT-C20)**							
Linoleic acid (C18:2)	-0.988***						
Palmitic acid (C16:0)	-0.838***	0.841***					
Stearic acid (C18:0)	-0.159	0.038	-0.106				
Arachidic acid (C20:0)	-0.174	0.045	-0.126	0.969***			
Gadoleic acid (C20:1)	0.433***	-0.348**	-0.303**	-0.828***	-0.791***		
Behenic acid (C22:0)	-0.132	0.038	-0.172	0.320	0.486**	-0.067	
Lignoceric acid (C24:0)	0.184	-0.147	-0.216*	-0.592**	-0.485**	0.790***	0.251*

*, **, and *** denotes significance level at 0.05%, 0.01% and 0.001%, respectively.

### QTL identification for different fatty acids

**Fig 3:**
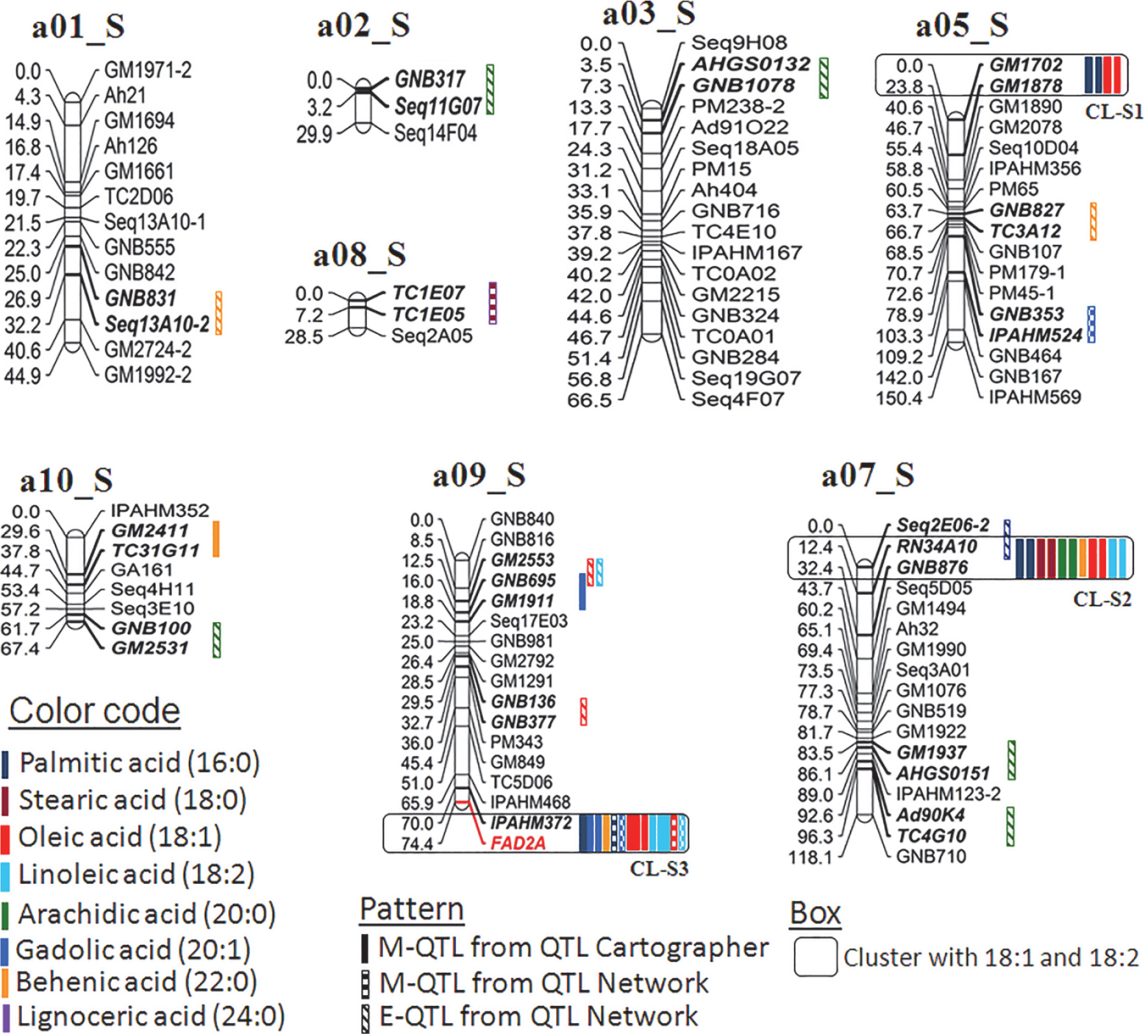
Genetic map of the A-genome of the S-population showing main-effect (M-QTLs) and epistatic (E-QTLs) QTLs for different fatty acids. This figure shows positions of M-QTLs detected by QTLCartographer and QTLNetwork while E-QTLs detected by QTLNetwork on the peanut A-genome for six fatty acids namely palmitic acid (C16:0), stearic acid (C18:0), arachidic acid (C20:0), gadoleic acid (C20:1), behenic acid (C22:0), and lignoceric acid (C24:0). Also M-QTLs and E-QTLs identified for oleic acid (C18:1) and linoleic acid (C18:2) from a previous study [24] were also plotted to draw comparison with the present study.

**Fig 4:**
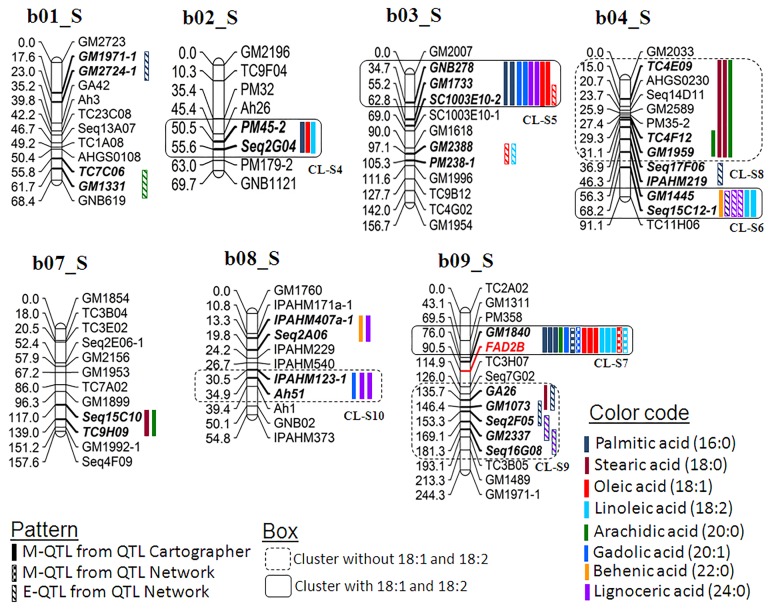
Genetic map of the B-genome of the S-population showing main-effect (M-QTLs) and epistatic (E-QTLs) QTLs for different fatty acids. This figure shows positions of M-QTLs detected by QTLCartographer and QTLNetwork while E-QTLs detected by QTLNetwork on the peanut B-genome for six fatty acids namely palmitic acid (C16:0), stearic acid (C18:0), arachidic acid (C20:0), gadoleic acid (C20:1), behenic acid (C22:0), and lignoceric acid (C24:0). Also M-QTLs and E-QTLs identified for oleic acid (C18:1) and linoleic acid (C18:2) identified from a previous study [24] were also plotted to draw comparison with the present study.

**Fig 5:**
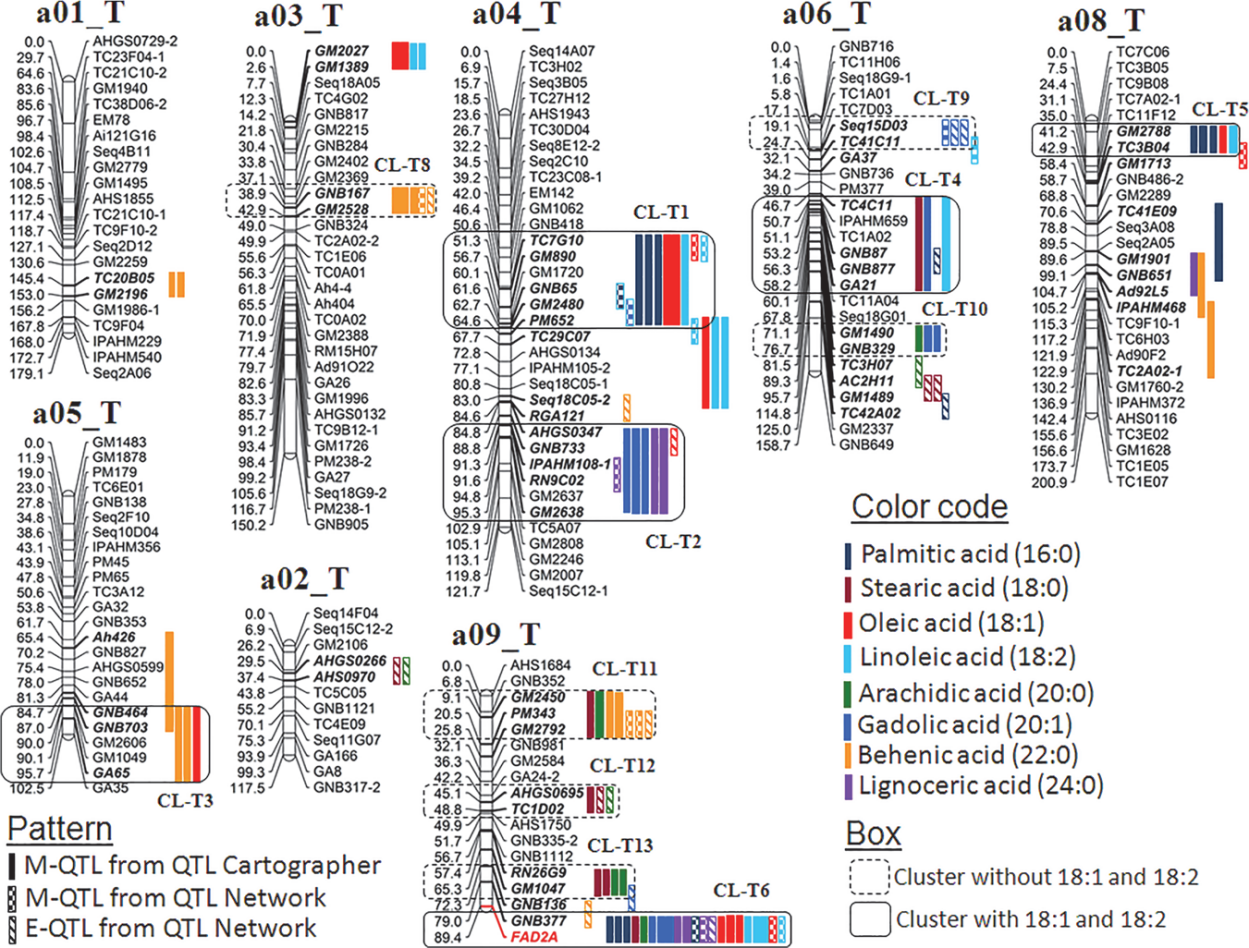
Genetic map of the A-genome of the T-population showing main-effect (M-QTLs) and epistatic (E-QTLs) QTLs for different fatty acids. This figure shows positions of M-QTLs detected by QTLCartographer and QTLNetwork while E-QTLs detected by QTLNetwork on peanut A-genome for six fatty acids, palmitic acid (C16:0), stearic acid (C18:0), arachidic acid (C20:0), gadoleic acid (C20:1), behenic acid (C22:0), and lignoceric acid (C24:0). Also M-QTLs and E-QTLs identified for oleic (C18:1) and linoleic acid (C18:2) identified from a previous study [24] were also plotted to draw comparison with the present study.

**Fig 6:**
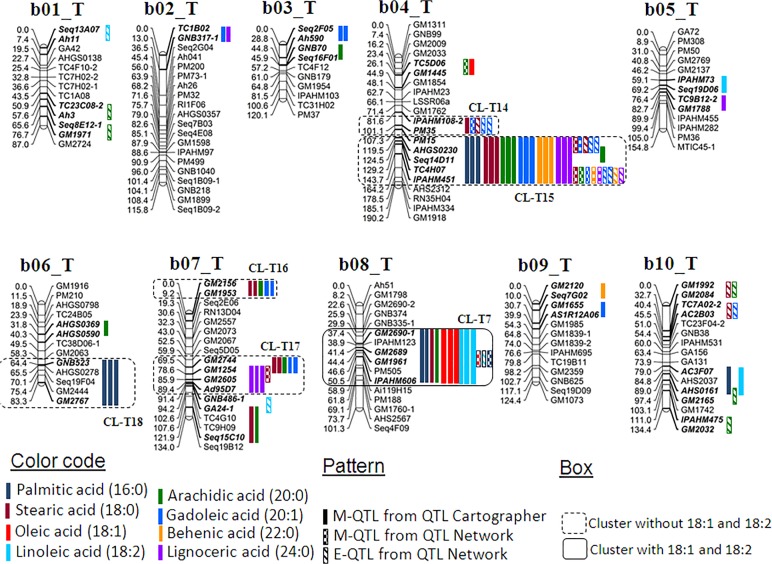
Genetic map of the B-genome of the T-population showing main-effect (M-QTLs) and epistatic (E-QTLs) QTLs for different fatty acids. This figure shows positions of M-QTLs detected by QTLCartographer and QTLNetwork while E-QTLs detected by QTLNetwork on peanut B-genome for six fatty acids namely palmitic acid (C16:0), stearic acid (C18:0), arachidic acid (C20:0), gadoleic acid (C20:1), behenic acid (C22:0), and lignoceric acid (C24:0). Also M-QTLs and E-QTLs identified for oleic (C18:1) and linoleic (C18:2) acid identified from a previous study [24] were also plotted to draw comparison with the present study.

Multi-season phenotypic data together with genotypic data generated on two mapping populations were used for conducting QTL analysis. The QTL analysis using QTLCartographer and QTLNetwork resulted in identification of 164 main effect QTLs (M-QTLs) and 27 epistatic QTLs (E-QTLs) for the six fatty acid traits in both populations (Tables 2, 3 and 4). The phenotypic variation explained (PVE) for these QTLs ranged from 0.16% (T_mqPA_b08-2) to the maximum of 40.56% (T_mqSA_b04-1) (S1–S5 Table). All the 55 QTLs (M-QTLs and E-QTLs) were mapped across 29 QTLs of 15 linkage groups in the S-population while 136 QTLs on 18 linkage groups in the T-population. The genomic locations of the above mentioned QTLs were shown on the A-genome and B-genome of the S- (Figs 3 and 4) and the T-population (Figs 5 and 6). The QTLs with >10% PVE have been referred as “major QTLs” while the QTLs with <10% PVE have been referred as “minor QTLs”. Furthermore, if the same QTL has been detected in more than one year at the same location, this has been referred as a “consistent QTL”.

**Table 2:** Summary of main-effect QTLs (M-QTLs) detected by QTLCartographer in the S- and T-population.

Traits	S-population				T-population			
	QTLs identified	LOD value range	Phenotypic variance (%)	Additive effect (a0)	QTLs identified	LOD value	Phenotypic variance (%)	Additive effect (a0)
Palmitic acid (16:0)	11	2.50–17.22	1.7–22.04	2.48 to (-) 1.09	19	2.54–23.92	3.06–37.37	0.358 to (-) 0.260
Stearic acid (18:0)	6	2.56–4.17	3.26–5.90	(-) 0.127 to (-) 0.299	15	2.51–21.75	2.63–40.57	0.589 to (-) 0.155
Arachidic acid (20:0)	6	3.11–4.10	3.60–6.40	0.057 to (-) 0.144	14	2.55–22.28	3.05–36.93	0.192 to (-) 0.065
Gadoleic acid (20:1)	7	2.55–5.55	2.55–8.77	0.107 to (-) 0.176	20	2.68–15.11	2.98–15.11	0.137 to (-) 0.054
Behenic acid (22:0)	5	2.52–4.41	2.88–6.95	0.374 to (-) 0.225	16	2.54–8.08	4.74–13.56	0.150 to (-) 0.099
Lignoceric acid (24:0)	5	2.53–5.27	2.89–6.58	0.219 to (-) 0.151	13	2.56–6.23	3.85–12.61	0.091 to (-) 0.048
Total	40	2.50–17.22	1.7–22.04	2.48 to (-) 0.144	97	2.51–23.92	2.63–40.57	0.589 to (-) 0.048

**Table 3:** Summary of main-effect QTLs (M-QTLs) detected by QTLNetwork in the S- and T-population.

Traits	S-population				T-population			
	QTLs identified	P value range	Phenotypic variance (%)	Additive effect (a0)	QTLs identified	P value range	Phenotypic variance (%)	Additive effect (a0)
Palmitic acid (16:0)	2	0	8.55–12.55	0.722–0.943	3	0.00	0.16–22.58	0.183 to (-) 0.586
Stearic acid (18:0)	1	3.2xE-5	2.71	0.1007	4	0.00	0.72–28.93	0.188 to (-) 0.314
Arachidic acid (20:0)	NIL	NIL	NIL	NIL	3	0.00 to (-) 1.1xE-5	4.38–28.93	0.038 to 0.105
Gadoleic acid (20:1)	3	0	3.26–5.66	(-) 0.062 to (-) 0.111	5	0.00 to 0.011	2.93–16.34	0.084 to (-) 0.039
Behenic acid (22:0)	NIL	NIL	NIL	NIL	3	0.00 to 3xE-5	4.89–9.44	0.107 to 0.153
Lignoceric acid (24:0)	NIL	NIL	NIL	NIL	3	0.00	3.79–4.50	0.055 to (-) 0.072
Total	6				21			

**Table 4:** Summary of epistatic QTLs (E-QTLs) detected by QTLNetwork in the S- and T-population.

Traits	QTLs identified	PV (%) range	AA range	SE range	P-Value range
**S-population**	** **	** **	** **	** **	** **
Palmitic acid (16:0)	3	1.57–3.65	0.567 to (-) 0.423	0.084 to 0.105	0.984 to 0.999
Stearic acid (18:0)	NIL	NIL	NIL	NIL	NIL
Arachidic acid (20:0)	3	2.91–5.53	(-) 0.056 to (-) 0.038	0.008 to 0.009	0.613 to 0.956
Gadoleic acid (20:1)	NIL	NIL	NIL	NIL	NIL
Behenic acid (22:0)	1	4.51	-0.0973	0.018	0.985
Lignoceric acid (24:0)	2	3.52–5.06	0.062 to (-) 0.072	0.011 to 0.016	0.979 to 0.989
**T-population**	** **	** **	** **	** **	** **
Palmitic acid (16:0)	1	5.67	0.3164	0.0487	0
Stearic acid (18:0)	4	1.78–6.84	0.17 to (-) 0.144	0.047–0.059	0.00 to 3E-4
Arachidic acid (20:0)	4	3.07–8.12	0.264 to (-) 0.795	0.011–0.159	0.00 to 7E-5
Gadoleic acid (20:1)	5	0.11–3.1	0.088 to (-) 0.0571	0.0135–0.0186	0.00 to7E-6
Behenic acid (22:0)	3	1.24–5.33	0.122 to (-) 0.845	0.021–0.025	0.00 to 0.01
Lignoceric acid (24:0)	1	1.06	0.0394	0.0161	0.01408

Of the total 164 M-QTLs identified, 35 M-QTLs were major QTLs. In the case of the S-population, three major QTLs were identified using QTLCartographer (S1 Table) while one major QTL with QTLNetwork (S3 Table). Interestingly, all these four major QTLs were detected for palmitic acid located on linkage group b09_S (S1 and S3 Tables; Figs 3 and 4). In contrast, the T-population had 22 and 9 major QTLs using QTLCartographer (S2 Table) and QTLNetwork (S4 Table), respectively. Among the 22 major QTLs identified by QTLCartographer, there were at least two major QTLs associated with each of these six fatty acids (S2 Table). The remaining nine major QTLs were associated with four fatty acids i.e., palmitic acid (three QTLs), arachidic acid (three QTLs), behenic acid (one QTL) and lignoceric acid (two QTLs) (S4 Table).

### Main-effect QTLs (M-QTLs) for different fatty acids

For palmitic acid (16:0), a total of 11 and 19 M-QTLs were identified using QTLCartographer in the S- and T-population with PVE up to 22.04% and 37.37%, respectively (Table 2). Only one consistent QTL (GM1840-*FAD2B*) was located on the linkage group b09_S in the S-population (Figs 3 and 4). The ‘NC94022’ alleles had higher additive effect and played important role in increasing palmitic acid (Figs 3 and 4; S1 Table). Similarly in the T-population, four consistent QTLs were detected in the marker intervals of TC7G10-PM652, GNB377-*FAD2A*, PM15-IPAHM451, and GNB523-GM2767 on the linkage group a04_T, a09_T, b04_T, and b06_T, respectively (Figs 5 and 6). In this population, the GT-C20 alleles contributed to the high palmitic acid in all the identified QTLs (Figs 5 and 6; S2 Table). Further, a total of five M-QTLs were identified for palmitic acid using QTLNetwork i.e., two M-QTLs in the S-population and three M-QTLs in the T-population (Table 3, S2 and S4 Tables). One major M-QTL each for S- and T-population was identified on b09_S (S_mqPA_b09-3 between marker GM1840-*FAD2B*) and a09_T (T_mqPA_a09-2 between marker GNB377-*FAD2A*) explaining 12.44% and 22.58% PV, respectively (Figs 3, 4, 5 and 6). The parent genotype ‘NC94022’ contributed to higher palmitic acid in the S-population while ‘GT-C20’ did in the T-population (S2 and S4 Tables).

For stearic acid (18:0), six and 15 M-QTLs were detected using QTLCartographer in the S- and T-population, respectively (Table 2). Neither any consistent QTL nor any major QTL were detected for stearic acid in the S-population (S1 Table). The highest PVE could only be explained by the M-QTL ‘S_mqSA_a07-1’ i.e., 5.9% PVE. However in the T-population, there were three major QTLs with PVE of more than 25%. The M-QTL ‘T_mqSA_b04-1’ had the highest PVE of 40.56% mapped on the linkage group b04_T. One consistent QTL was identified on the linkage group b04_T (PM15-IPAHM451) with higher contribution from ‘Tifrunner’ (S2 Table; Fig 6). Further, four M-QTLs were identified in T-population using QTLNetwork, and three of these were major QTLs located on the linkage group b04_T with PVE of more than 17.8% (Table 3; S4 Table; Fig 6). In the S-population, however, only one M-QTL was detected between marker interval ‘TC1E07-TC1E05’ on the linkage group a08_S explaining only upto 2.71% PV (S3 Table; Fig 3).

For arachidic acid (20:0), a total of 6 and 14 M-QTLs were detected in the S- and T-populations using QTLCartographer. No major QTL was detected in the S-population and the QTL with the highest PVE was ‘S mqAA b09’ with 6.43% PVE located on the linkage group b09_S (S1 Table; Fig 4). One consistent QTL (TC4F12-GM1959) was identified on the linkage group a04_S with effect from the parent genotype ‘SunOleic 97R’. In the T-population, there were three major QTLs with > 29% PVE (S2 Table), and the highest PVE of 36.92% was explained by the QTL ‘T_mqAA_b04-1’ located on the linkage group b04_T (Fig 2). It was interesting to note that the consistent QTL identified on the linkage group b04_T (PM15-IPAHM451) for arachidic acid was the same as in case of stearic acid (S2 Table; Fig 6). Using QTLNetwork, three M-QTLs were identified in the T-population (Table 4) located on the same linkage group i.e., b04_T (Fig 6). Two of these M-QTLs contributed more than 28% PVE (S4 Table). No M-QTL was detected for arachidic acid in the S-population using QTLNetwork (S3 Table).

For gadoleic acid (20:1), QTLCartographer identified a total of 20 M-QTLs in the T-population with PVE ranging from 2.98% (T_mqGA_b07-4) to 26.13% (T_mqGA_b04-1) (S2 Table). Of these 20 M-QTLs, six were major QTLs and four were consistent QTLs on the linkage group a04_T (AHGS0347-GM2638), a09_T (GNB377-*FAD2A*), b04_T (PM15-IPAHM451) and b07_T (GM1254-GM2744) (Figs 5 and 6; S2 Table). The parent genotype ‘GT-C20’ contributed to the higher trait value in the consistent QTLs detected on the linkage group b07_T (GM1254-GM2744). In the S-population, although seven M-QTLs were identified, none of them were neither major QTLs nor were consistent. The highest PV (8.77%) was explained by the QTL ‘S_mqGA_b09-1’ mapped on the linkage group b09_S (Fig 4; S1 Table). The QTLNetwork detected a total of three and five M-QTLs in the S- and T-population, respectively (Table 3). All the three M-QTLs detected in the S-population were located on the different linkage groups (a05_S, a09_S and b09_S) with PVE ranging from 3.26% (S_mqGA_a05) to 5.66% (S_mqGA_a09-4). In contrast, three of the five M-QTLs detected in the T-population were major QTLs and were located on the linkage group b04_T (Table 3; S4 Table; Fig 6).

For behenic acid (22:0), the QTLCartographer identified a total of five minor M-QTLs in the S-population (Table 2, S1 Table). Similar to stearic acid and gadoleic acid, neither any consistent QTL nor any major QTL was detected for behenic acid in the S-population (S1 Table). In the T-population, total 16 M-QTLs were identified and two of these were major QTLs (S2 Table). There were two consistent QTLs identified on the linkage group a03_T (GNB167-GM2528), and b04_T (PM15-IPAHM451). The source for the higher additive effect alleles was ‘Tifrunner’ for both the consistent QTLs (S2 Table). Further, QTLNetwork software identified three M-QTLs in the T-population while none in the S-population (Table 3, S3 Table). These three M-QTLs were located on the linkage group a03_T (T_mqBA_a03-2), a09_T (T_mqBA_a09-1) and b04_T (T_mqBA_b04-2) with PVE up to 9.44% (Fig 6; S4 Table).

For lignoceric acid (24:0), total five and 13 M-QTLs were identified in the S- and T-populations, respectively using QTLCartographer (Table 2). In the S-population, PVE ranged from 2.88% (S_mqLA_b03-1) to 6.57% (S_mqLA_b08-2) (S1 Table). It was interesting to note that neither any consistent QTL nor any major QTL was detected for lignoceric acid in the S-population i.e., similar to stearic acid, gadoleic acid and behenic acid (S1 Table). In the T-population, of the total 13 M-QTLs, there were three major QTLs with PVE up to 12.61% (S2 Table). Two consistent QTLs namely PM15-IPAHM451 and GM1254-Ad95D7 were identified on linkage groups on b04_T and b07_T, respectively (Fig 6). The parent genotype ‘GT-C20’ was the source for higher additive effect contributor for consistent QTLs identified on the linkage group b04_T and b07_T (S2 Table). Further, three M-QTLs were identified using QTLNetwork in the T-population (Table 3) present on the linkage group a04_T, a09_T and b04_T (Figs 5 and 6) with maximum PVE of 4.5% (T_mqLA_b04-3). No M-QTL was detected in the S-population using QTLNetwork (S3 Table).

### Epistatic effect QTLs (E-QTLs) for different fatty acids

The QTLNetwork detected a total of 27 E-QTLs i.e., nine in the S-population and 18 in the T-population with only two-locus interactions (Table 4). The PVE detected in the S-population ranged from 1.57% to 5.53% and the additive effects of the two-locus interaction varied from 0.56 to (-) 0.42. Similarly in the T-population, the PVE varied from 0.11% to 8.12% and the two-locus additive effects varied from 0.31 to (-) 0.84. All the six fatty acids were found to be associated with at least one E-QTL identified in the T-population while four fatty acids in the S-population (Table 4, S5 Table). The two E-QTLs with more than one interaction were identified between GM1489-AC2H11 and TC41C11-Seq15D03 for stearic acid and gadoleic acid, respectively. Both of these E-QTLs were located on the linkage group a06_T. In the S-population, although there was no E-QTL identified for stearic acid and gadoleic acid but at least one E-QTL was detected for other four fatty acids (Table 4).

### QTLs controlling saturated and unsaturated fatty acids

In the light of *FAD2A* and *FAD2B* genes playing important role in controlling the two unsaturated fatty acids i.e., oleic acid (C18:1) and linoleic acid (C18:2) in the peanut oil, it would be interesting to compare the QTLs associated with the five SFAs and a UFA (gadoleic acid) (C20:1) with oleic and linoleic acid. In order to draw such comparison, QTLs identified by Pandey et al. [24] for oleic acid (C18:1) and linoleic acid (C18:2) were used for comparison with the QTLs identified for these six fatty acids in the present study. The S-population segregates for both *FAD2A* and *FAD2B* genes while the T-population segregates only for *FAD2A* gene. Therefore, an effort was made to compare the QTLs obtained for oleic acid and linoleic acid in the S- and T-population with the QTLs obtained for these minor fatty acids (palmitic, stearic, arachidic, gadoleic, behenic, and lignoceric) including clustering and localization in the same location (Figs 3, 4, 5 and 6).

In the S-population (Figs 3 and 4, box with solid line), a total of 15 M-QTLs and two E-QTLs identified for oleic acid (C18:1), and 13 M-QTLs and one E-QTL for linoleic acid (C18:2) identified by Pandey et al. [24] were used for comparison with QTLs identified for other minor fatty acids. By comparing the locations of these QTLs on the linkage groups, a genomic region on the genetic map was defined as a cluster (CL) if this region harbored at least three QTLs for different traits. By this measurement, total seven clusters (CLs) were identified wherein various minor fatty acid QTLs (identified in this study) as well as oleic acid and/or linoleic acid were co-localized in the same location of linkage group (Figs 3 and 4, box with solid line). Of these seven clusters (CL-S1 to CL-S7), oleic acid and linoleic acid were co-localized in four clusters (CL-S2, S3, S4 and S7) along with other fatty acids. The remaining three clusters (CL-S1, S5 and S6) had either oleic acid or linoleic acid. With respect to the genome-wide distribution of these clusters, three clusters were present on the A-genome (a05_S, a07_S and a0_S 9) (Fig 3) while four clusters on the B-genome (b02_S, b03_S, b04_S and b09_S) (Fig 4).

Similarly in the T-population (Figs 5 and 6, box with solid line), a total of 19 QTLs (18 M-QTLs and one E-QTL) for oleic acid whereas 20 QTLs (19 M-QTLs and one E-QTL) for linoleic acid identified by Pandey et al. [24] were compared in this study with other minor fatty acids. The analysis also resulted in identification of seven clusters (CL-T1 to CL-T7) in which oleic acid and/or linoleic acid co-localized along with other fatty acids across six linkage groups (Figs 5 and 6). Of these seven clusters, oleic acid and linoleic acid were co-localized in four clusters along with other fatty acids (CL-T1, T5, T6 and T7), while the other three clusters (CL-T2, T3 and T4) had either oleic acid or linoleic acid. Only one cluster was present on the B-genome (b08_T) (Fig 6) while the remaining six clusters were present on the A-genome (a04_T, a05_T, a06_T, a08_T and a09_T) (Fig 5).

### Clustering of the QTLs on the genome for different fatty acids

It was observed that many M-QTLs as well as E-QTLs were clustered together in the same linkage group for the minor fatty acids in this study. It was interesting to compare these clustered QTLs without any influence of QTL/gene/mutant alleles controlling oleic acid and linoleic acid, thereby suggesting an independent role in fatty acid metabolism.

In the S-population (Figs 3 and 4, box with dashed line), a total of three clusters (CL_S8 to CL_S10) were identified on three different linkage groups (b04_S, B08_S, and B09_S) present in the B-genome (Fig 4). No cluster was detected in A-genome. Cluster CL-S8 (TC4E09-GM1959) was identified on the linkage group b04_S which contained four M-QTLs i.e., two each for stearic acid and arachidic acid. The second cluster CL-S9 (GA26-Seq16G08) was identified on the linkage group b09_S and possessed four E-QTLs and one M-QTL each for palmitic acid, stearic acid, and lignoceric acid. The third cluster CL-S9 was identified on the linkage group b08_S between marker IPAHM123-1-Ah51 harboring three M-QTLs i.e., two for lignoceric acid and one for gadoleic acid.

Similarly, in the T-population (Figs 5 and 6, box with dashed line), a total of 11 clusters (CL_T8 to T18) were identified across six linkage groups. Six of the 11 clusters were located on the three linkage groups (a03_T, a06_T and a9_T) of the A-genome (Fig 5) and five clusters were located on three linkage groups (b04_T, b06_T and b07_T) of the B-genome (Fig 6).

## Discussion

Fatty acid composition of peanut oil affects overall oil quality related parameters such as nutrition and shelf life. Generally, high % of oleic acid and low % of linoleic acid (high oleic / linoleic acid ratio) in the cooking peanut oil is healthier for human consumption as it reduces the risk of cardiovascular disease (CVD) by lowering low-density lipoproteins (LDL) levels in blood in addition to providing longer shelf life and oxidative stability to the peanut products [3, 4, 5]. Although, oleic acid and linoleic acid together makes about 80% of the total fatty acids in peanut oil, the nutritional quality of minor fatty acids which comprises of the rest 20% fatty acids can’t be ignored [7]. The palmitic acid was reported to increase the risk for multiple life-threating diseases such CVD and atrial fibrillation. Stearic acid was found to lower the risk of atrial fibrillation and has neutral effect on blood LDL [10, 11]. Based on the information on the effect of different fatty acids to the human health and shelf life of the peanut products, it is clear that improved cultivars with right combination of fatty acids needs to be developed. The peanut oil with minimum saturated fatty acids (SFAs) and polyunsaturated fatty acids (PUFAs) are preferred considering the health benefits associated with oil consumption. This reduction can be achieved to a limited extent by increasing the quantity of monounsaturated fatty acids (MUFAs). The efforts in this direction could only be initiated upon availability of genomics tools for selecting right combination of genes/alleles for the right combination of phenotypes for different fatty acids. Therefore, altering peanut oil fatty acid composition has the prospect to increase the nutritional quality of peanut products. In this context, the present study was conducted to identify QTLs controlling different fatty acids in the peanut oil. The information generated through this study will further improve our understanding on genetic control of different fatty acids and will help in manipulating the composition for a desirable phenotype for different fatty acids.

### Fatty acid composition and phenotypic correlation between different fatty acids

Phenotypic analysis of the multiseason phenotyping data revealed considerable phenotypic variation for different fatty acids in both the mapping populations (Figs 1 and 2). It is important to note that the S-population segregates for two recessive mutant genes (*FAD2A* and *FAD2B*) while the T-population segregates only for *FAD2A*. As these two mutant genes are known to affect the oleic acid and linoleic acid content, correlation study revealed that these two major fatty acids also affect the minor fatty acids. A positive correlation was observed for oleic acid with gadoleic acid and lignoceric acid while a negative correlation with stearic, palmitic, linoleic, arachidic and behenic acids (Table 1). This is in congruence with the results obtained in the previous studies in peanuts [2, 15] as well as in a wide range of other crops such as palm oil [25], flax [26], maize [27] and brassica [18].

**Fig 7:**
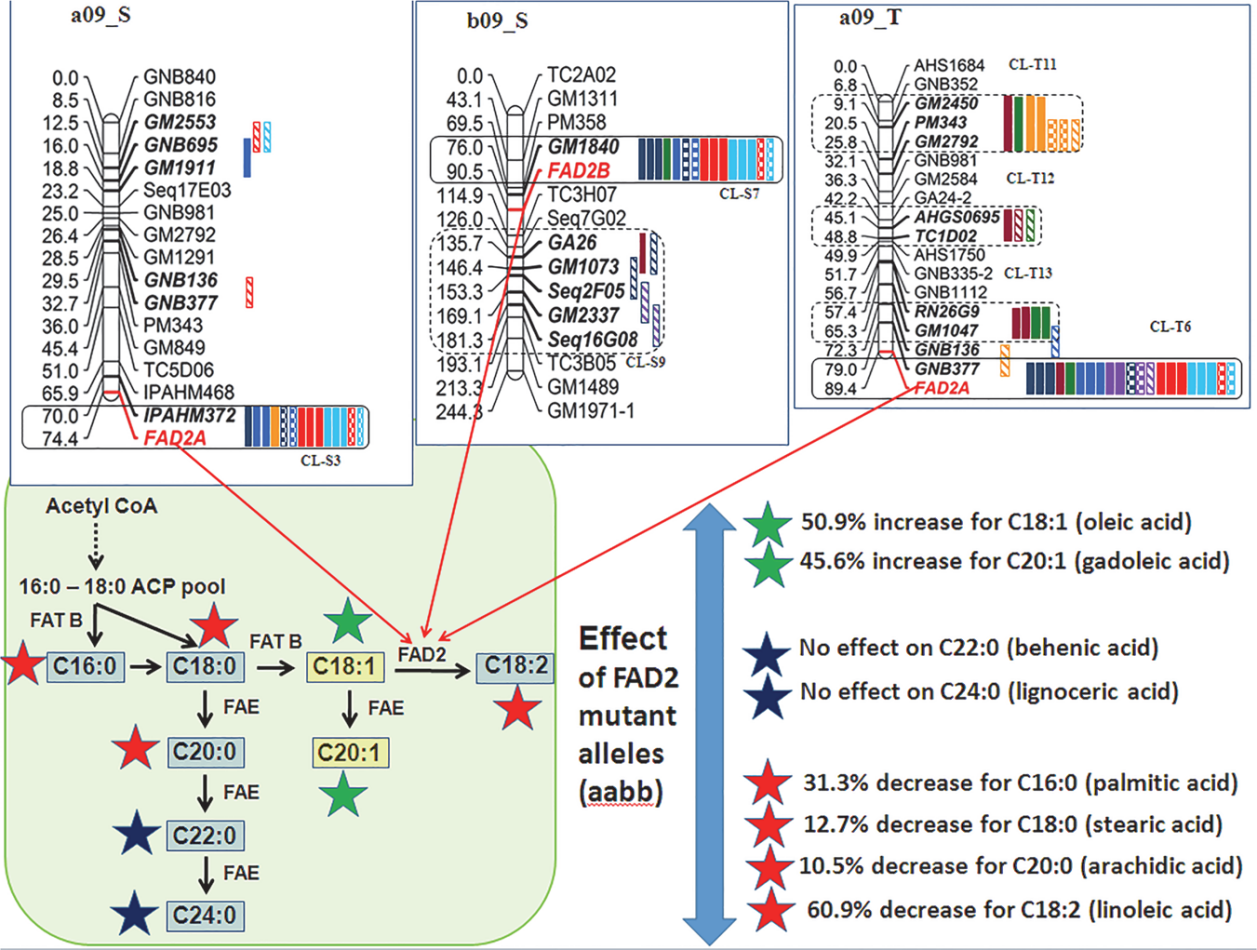
Simplified schematic part of the fatty acid pathway known in plants. Blue shaded boxes indicate probable negatively correlated pathway while yellow boxes indicate positively correlated pathway due to presence of mutant FAD2 genes. Action of mutant *FAD2A* and *FAD2B* in the S-population while *FAD2A* QTL in the T-population stops desaturation of oleic acid (C18:1) to linoleic acid (C18:2). The capital letters in parenthesis (A-D) indicate the pathway through which fatty acids are produced by acyl carrier protein (ACP) synthesis within the plastid. ACCase, Acetyl-CoA carboxylase; FAS, fatty acid synthase; KAS, ketoacyl ACP synthetase; Δ9DES, Δ9 desaturase; ACS, acyl-CoA synthetase; FAT B, palmitoyl-ACP thioesterase; FATA, stearoyl-ACP thioesterase; FAE, fatty acid elongase, *FAD2*, fatty acid desaturase.

The correlation between different fatty acids can be better understood if current understanding of biosynthetic pathway of fatty acid metabolism in plants is taken under perspective (Fig 7). In plants, the enzyme acetyl CoA play a significant role in formation of different types of fatty acids such as SFAs, MUFAs and PUFAs. Usually the first double bond is inserted between ninth and tenth carbon from the carboxyl end (Δ^9^) which forms the palmitic acid (C16:0). The addition of acetyl residues makes stearic acid (C18:0). Although there is no change to the palmitic acid but the formation of stearic acid gave rise to two directions. The first direction upon addition of acetyl residue reactions forms arachidic acid (C20:0), followed by behenic acid (C22:0) and then lignoceric acid (C24:0). The second direction upon desaturation of stearic acid (C18:0) produces oleic acid (18:1) which possess one double bond. Further, the oleic acid (C18:1) can either produce linoleic acid (C18:2, by desaturation) or gadoleic acid (20:1, by addition of acetyl residues) [17, 18, 19]. This is a known fact that the *FAD2* genes encode for microsomal oleoyl-PC desaturase or Δ^12^ fatty acid desaturase [20] i.e. formation of C18:2 from C18:1, the mutant allele for *FAD2* basically blocks this pathway. Plants however cannot afford to completely eliminate the synthesis of linoleic acid (C18:2) as linoleic acid further forms linolenic acid (C18:3) which plays an essential role in photosynthesis [28] and pollen development [29]. Therefore, we hypothesize that in genotypes with mutant *FAD2* genes might trigger the plant to mobilize its resources to the production of more linoleic acid, thereby resulting in the accumulation of oleic acid (C18:1) while burning up of the source, in this case palmitic acid (C16:0) (Fig 7). This phenomenon would have led to increase in gadoleic acid (C20:1) content which is formed from oleic acid (C18:1). On the other hand since more palmitic acid (C16:0) is mobilized towards the oleic acid (C18:1), it led to decease in the content of palmitic acid (C16:0), stearic acid (C18:0), arachidic acid (C20:0), behenic acid (C22:0) and lignoceric acid (C24:0). This could be the reason for the observed positive correlation of oleic acid (C18:1) with gadoliec acid (C20:1), while negative correlation with palmitic acid (C16:0), stearic acid (C18:0), arachidic acid (C20:0), behenic acid (C22:0) and lignoceric acid (C24:0). The information on the above correlations between different fatty acids will be very useful while selecting the particular fatty acid for genetic manipulation to get desired phenotypes.

### QTLs for different fatty acids determining oil quality

The identification of QTLs controlling oil-quality traits should contribute to better understanding of oil synthesis as well as use of linked markers will facilitate oil quality improvement through genomics-assisted breeding. It is noteworthy here that this is the first study to report the identification of QTLs for the minor fatty acids in peanut. In the present study, two genetic software were employed for identification of main-effect QTLs (M-QTLs) and one for epistatic QTL interaction (E-QTL). Thus the total phenotypic variation for a complex trait is the result of multiple QTLs as well as their interactions. QTL analysis resulted in the identification of a total of 191 QTLs (164 M-QTLs and 27 E-QTLs) for the six fatty acids in the S- and T-population (Tables 2, 3 and 4) across 15 linkage groups with 29 QTLs in the S-population while 18 linkage groups in the T-population with 45 QTLs. The PVE ranged from 0.16% to 40.56% indicating role of minor as well as major QTLs in controlling fatty acids. Large number of QTLs (34 M-QTLs) with direct effect were major QTLs as they accounted for over 10% of PVE and were located on four homeologous chromosomes in the A- and the B-genome (a04, a09, b04 and b09). This indicates that these two homeologous linkage groups are the home for major QTLs which play a significant role in controlling peanut oil fatty acid composition.

The number of QTLs identified in the T-population (118 M-QTLs and 18 E-QTLs) was more than double that of in the S-population (46 M-QTLs and nine E-QTLs) indicating that genetic control of minor fatty acids is more complex in the T-population than in the S-population. The above difference also might be because of absence of *FAD2B* mutant allele in the T-population which had shown control over several fatty acids in the S-population such as oleic acid, linoleic acid and palmitic acid. In addition to the above possible explanation, genetic background might have also played an important role in the formation of these fatty acids. Similarly, when the total numbers of M-QTLs in both S- and T-population for these minor fatty acids were taken into account, it revealed altogether a different picture. A large number of M-QTLs were identified for palmitic acid (35) and gadoleic acid (35), followed by stearic acid (26), arachidic acid (23), behenic acid (24) and lignoceric acid (21). The higher number of QTLs for each minor fatty acid indicates that many other genetic factors are also involved in fatty acid metabolism. However, E-QTLs from these two populations indicated that the highest E-QTLs were associated with arachidic acid (7), gadoleic acid (5), palmitic acid (4), stearic acid (4), behenic acid (4) and lignoceric acid (3). Therefore, there were several epistatic QTLs which could also control these traits, such as in soybean [30]. A total of three E-QTLs were identified in soybean for linolenic acid [30], and there is no other report on identification of E-QTLs in any crops for the traits studied in this study.

### QTL clusters on the genome for different fatty acids

*FAD2* genes play a critical role in modifying the overall composition of fatty acids in peanut oil and the effect of these genes have been well established [24]. Further curiosity was to see if these genes also had effect on other fatty acids i.e., other than oleic acid and linoleic acid. The other interest was to identify important QTLs including clusters whether they play a significant role in the synthesis of other fatty acids. Therefore, the emphasis was to look for other QTLs which control these minor fatty acids besides the *FAD2* genes. In order to extract above mentioned information, the M-QTLs and E-QTLs identified for oleic acid and linoleic acid in the same two populations by Pandey et al. [24] were plotted along with the M-QTLs and E-QTLs detected for these six minor fatty acids in the present study. Plotting of QTLs onto the genetic maps revealed a total of 10 multiple QTL clusters in the S-population and 18 in the T-population. Interestingly in these two populations, seven clusters co-localized together for oleic acid and/or linoleic acid along with other minor fatty acids. The independent clusters without oleic and linoleic acids were three in the S-population and 11 in the T-population (Figs 3, 4 and 5). It has been known from the previous studies that when the two mutant genes are present together, the oleic acid content is much higher than in the genotype possessing only one gene [15, 24]. In summary, the genetic control of the fatty acids in the S-population is much simpler than that in the T-population; and this could be due to the S-population possessing two recessive genes of *FAD2A* and *FAD2B* and the T-population has only one i.e., *FAD2A*.

Allelic analysis revealed that in the S-population, out of the total seven clusters co-located for oleic acid and/or linoleic acid, four clusters had higher trait alleles from only one parent i.e. ‘SunOleic 97R’ or ‘NC94022’, while the other three clusters had higher alleles from different parents for different traits which co-localized in a particular cluster. In the cluster CL-S3 on linkage group a09_S (Fig 3) between marker IPAHM372-*FAD2A*, ‘SunOleic 97R’ increased oleic acid and gadoleic acid traits, in contrast ‘NC94022’ increased palmitic acid, linoleic acid and behenic acid. This indicates that the contrasting alleles from ‘SunOleic 97R’ decreased palmitic acid, linoleic acid and behenic acid. This can be correlated with the fact that ‘SunOleic 97R’ as a parental line has high oleic acid content due to the presence of *FAD2A* mutant allele. Similarly, in the T-population, seven clusters were identified possessing co-localized QTLs for oleic acid and/or linoleic acid. However, only one cluster (CL-T2) had higher trait alleles from one parent, i.e. ‘Tifrunner’ while remaining six clusters had higher alleles from different parents for different traits. Additionally, 11 other clusters were identified to co-host QTLs for one and/or all six fatty acids. Out of these 11 clusters, six clusters had higher alleles from one parent while the remaining five clusters had contrasting allele effects for higher traits. The identification of QTL clusters is a common phenomenon where several related traits contributing the complex traits or part of same metabolism/pathway were studied. For example such clusters for drought tolerance related traits were identified where several component and related traits to drought tolerance were phenotyped and used for QTL analysis [31]. Similarly when several stages after disease infestation for rust and late leaf spot were phenotyped, the QTL analysis detected QTL clusters with co-localized QTLs for same as well as related traits [32, 33]. The other recent example was clustering of different QTLs for oleic, linoleic acid and oleic/linoleic acid ratio with *FAD2A* genes [24]. These clusters have a great relevance in genomics-assisted breeding as introgression of one such QTL can have phenotypic impact for other traits also for which QTLs were found co-localized on the same location. One such example has recently been seen in case of introgressing rust resistance QTL into elite peanut cultivars using MABC approach [34]. In this case the QTL for another disease, late leaf spot, was also present on the same location and was introgressed with the rust resistance. This resulted in the introgression of two foliar fungal diseases simultaneously in the genetic background of three elite cultivars [34]. Therefore, the QTL clusters may play important role in improving more than one fatty acid by transferring just one QTL cluster into the target cultivars.

### Insights on the genetic control of fatty acid synthesis pathway

The phenotyping of an array of fatty acids produced from the very complex fatty acid pathway in peanut has provided an opportunity to correlate different fatty acids. QTL analysis revealed several ‘major effect’ QTLs for these traits. As it was observed from the phenotyping data, QTL analysis has also supported the phenotypic correlation. These analyses not only resulted in the identification of QTLs controlling each fatty acid but also identified QTL clusters. The fatty acid biosynthetic pathway is highly complex which involves multiple steps and each step is carried out by a different enzymatic activity (Fig 7). For example, ketoacyl ACP synthetase (KAS) elongates palmitic acid and stearic acid, palmitoyl-ACP thioesterase (FATB) terminates elongation of palmitic acid, stearoyl-ACP thioesterase (FATA) terminates elongation of stearic acid, stearoyl-ACP desaturase (SAD) desaturates stearic acid to oleic acid, fatty acid desaturase (FAD2) desaturates oleic acid to linoleic acid, and fatty acid elongase (FAE) elongates both saturated as well as unsaturated C18 to C20, C22 and C24 [17, 18, 19]. The results from this study have brought insights on the genetic control of the pathway. Specially, the role of *FAD2* genes has well been analyzed for different fatty acids. It has been observed that if both the mutant alleles together increase the production of oleic acid and gadoleic acid while decreased the production of linoleic acid, palmitic acid, stearic and arachidic acid (Fig 7; Table 5). There was no effect of these mutant alleles on the behenic and lignoceric acid (Fig 7; Table 5). The information generated will now help in understanding the fatty acid synthetic pathway in better way. Nevertheless, further study on the role of other genetic factors is required to develop much better understanding of this pathway from the genetics and genomics aspect. In this context, genome sequencing effort for tetraploid peanut is in progress and Tifrunner is being used as the reference genome [22]. Further, all the remaining three parental genotypes are planned for re-sequencing along with the set of RILs which will provide much better information and thus, develop much better understanding of the pathway. Once the genome sequence is completed it would be interesting in the future to determine the homoeology of chromosomes from A- and B-sub-genomes; and better identify orthologous genes for most of these enzymes. Furthermore, the individual cluster would be unraveled thereby enhancing our understanding of fatty acid metabolism in peanut and pyramiding favorite alleles from different fatty acids by marker-assisted selection for improving peanut fatty acid composition.

**Table 5:** Phenotypic effect of *FAD2A* and *FAD2B* genes for oil quality traits in the S- and T-population.

Traits	S-Population				T-population	
	AABB (66)	AAbb (51)	aaBB (65)	aabb (60)	AABB (92)	aaBB (130)
Palmitic acid (C16:0)	12.43	10.71	10.84	8.54	11.27	10.01
Stearic acid (C18:0)	2.52	2.42	2.3	2.2	3.18	2.74
Arachidic acid (C20:0)	1.24	1.2	1.18	1.11	1.49	1.36
Gadoleic acid (C20:1)	1.03	1.17	1.22	1.5	1.06	1.3
Behenic acid (C22:0)	2.5	2.41	2.51	2.35	2.94	2.98
Lignoceric acid (C24:0)	1.26	1.28	1.33	1.35	1.5	1.61
Oleic acid (C18:1)*	46.52	57.03	55.58	70.23	44.23	52.56
Linoleic acid (C18:2)*	32.5	23.79	25.03	12.72	34.33	27.44

AA: wild A sub-genome allele for *FAD2A* gene in homozygous condition, aa: mutant A sub-genome allele for *FAD2A* gene in homozygous condition, Aa: *FAD2A* gene in heterozygous condition in A sub-genome, BB: wild B sub-genome allele for *FAD2B* gene in homozygous condition, bb: mutant B sub-genome allele for *FAD2B* gene in homozygous condition, Bb: *FAD2B* gene in heterozygous condition in B sub-genome. Since the RIL populations were genotyped and phenotyped, there should be no heterozygous Aa or Bb genotype.

*The phenotypic data taken for comparison from Pandey et al. [24].

## Materials and Methods

### Plant materials

Two RIL populations derived from the cross ‘SunOleic 97R’ × ‘NC94022’ (referred as S-population and the cross ‘Tifrunner’ × ‘GT-C20’ (referred as T-population) with 352 and 248 individuals, respectively, were used in this study [24, 35]. The ‘SunOleic 97R’ is a high oleic runner market-type peanut with two recessive mutant genes of *FAD2A* and *FAD2B* from the original high oleic line F435 [36] while ‘NC94022’ is a breeding selection from Virginia type and *hirsuta* with wild type *FAD2* alleles. The ‘Tifrunner’ is a runner market-type cultivar with only one mutant allele *FAD2A* [37], while ‘GT-C20’, a Spanish-type breeding line, has wild type *FAD2* alleles.

### Phenotyping of mapping populations by chemical analysis

The complete sets of the S- and T-population along with parental lines were grown in three replications in the field in 2010 and 2011 at the Bellflower Farm, Tifton, Georgia, USA.

The harvested pods from all the three replications were dried, packed and sent for the determination of fatty acid profiles to Plant Genetic Resources Conservation Unit, USDA-ARS, Griffin, USA. An Agilent 7890A (Agilent Technologies, Santa Clara, California, USA) gas chromatograph with a flame ionization detector (FID) was used to determine the fatty acid composition. Oil was extracted from ~150 mg of finely grounded peanut seed tissue in 5.0 ml of n-heptane (Fisher Scientific). Then it was trans-esterified to fatty acids methyl esters (FAME) with 500 μl of 0.5 N sodium methoxide (NaOCH3). After 120 minutes, organic layer was separated from the aqueous layer and seed residue by adding 7.0 ml of distilled water. The organic layer contains methyl esters; 1.5 ml of the aliquot was transferred to autosampler for gas chromatography (GC) analysis. Peak separation was done on a DB-225 capillary column (5 m × 0.25 mm id with a 0.25 μm film) from Agilent Technologies, Santa Clara, California, USA. At a split ration of 60:1, 1 μl of the sample was injected onto the column maintained isothermally at 280°C. The temperature for inlet was same as that of column while that of detector was set to 300°C. Helium was used as a carrier gas and was set to a flow rate of ~1.0 ml/min (38 cm/sec). A FAME standard mix RM-3 (Sigma-Aldrich, St. Louis, Missouri, USA) was used to establish peak retention times. Each sample took 12 mins for running. A total of six fatty acids palmitic acid (C16:0), stearic acid (C18:0), arachidic acid (C20:0), gadoleic acid (C20:1), behenic acid (C22:0), and lignoceric acid (C24:0) along with oleic acid (C18:1) and linoleic acid (C18:2) was measured in each peanut sample.

### Development and use of improved genetic maps

The DNA from each RIL was extracted from fresh leaves along with their parental genotypes as described in Qin et al. [35]. After assessing the quality and quantity of isolated genomic DNA, a total of 230 and 402 polymorphic markers were identified for S- and T-population, respectively. The first individual genetic maps for the S- and T-population were constructed with 172 (920.7 cM) and 236 (1,213.4 cM) marker loci, respectively, by Qin et al. [35]. Further genotyping data for 215 SSR loci in S-population and 390 SSR loci in T-population were generated on the full sets of RILs and used for construction of genetic maps as described in Pandey et al. [24]. The detailed development of the improved maps had been used in this study, which has 206 (1780.6 cM) and 378 (2487.4 cM) marker loci for the S- and the T-population, respectively.

### QTL analysis using QTLCartographer and QTLNetwork

All six fatty acids were used as traits for QTL mapping. Since the data was generated for two years, QTL analysis was performed for individual year (trait_2010, trait_2011) as well as the mean (trait_mean). Windows QTLCartographer version 2.5 [38] was used for identification of main-effect QTLs (M-QTLs); QTLNetwork version 2.0 [39] was used for M-QTL and epistatic QTL interactions (E-QTLs). For identification of location and effect of M-QTL using QTLCartographer composite interval mapping (CIM) approach was followed as per the parameters such as, LOD of 2.5, model 6, scanning intervals of 1.0 cM between markers and putative QTL with a window size of 10.0 cM, forward-backward stepwise regression as background control set by the number of marker cofactors, 500 permutations with 0.05 significance level and “Locate QTL” option. Mixed linear model was employed to identify M-QTL and E-QTL from QTLNetwork with single-dimension genome scan (with an option to map epistasis) and a two-dimensional genome scan to detect epistatic interactions with or without single-locus effect. For performing QTL analysis, factors such as 1000 permutations, genome scan (walk speed of 1.0 cM, testing window and filtration window size of 10.0 cM), experimental-wise significant level of 0.05 for detection of QTL with their effect and Monte Carlo Markov Chain for estimating QTL effects were selected.

## Supporting Information

S1 Table Main-effect QTLs (M-QTLs) identified for fatty acids by QTLCartographer in the S-population. This table shows details on location, flanking marker loci, LOD value, phenotypic variance explained and additive effects of 40 M-QTLs detected by QTLCartographer.
(XLSX)

S2 Table Main-effect QTLs (M-QTLs) identified for fatty acids by QTLCartographer in the T-population. This table shows details on location, flanking marker loci, LOD value, phenotypic variance explained and additive effects of 97 M-QTLs detected by QTLCartographer.
(XLSX)

S3 Table Main-effect QTLs (M-QTLs) identified for fatty acids by QTLNetwork in the S-population. This table shows details on location, flanking marker loci, phenotypic variance explained and additive effects of 6 M-QTLs detected by QTLNetwork.
(XLSX)

S4 Table Main-effect QTLs (M-QTLs) identified for fatty acids by QTLNetwork in the T-population. This table shows details on location, flanking marker loci, phenotypic variance explained and additive effects of 21 M-QTLs detected by QTLNetwork.
(XLSX)

S5 Table Epistatic QTLs (E-QTLs) identified for fatty acids by QTLNetwork in the S- and T-populations. This table shows details on search range, F value, phenotypic variance explained, number of interacting loci and names interacting loci for 27 E-QTLs detected by QTLNetwork.
(XLSX)
